# Social Media Use as a Factor Associated With the Unsupervised Use of Acne Treatment Products Among Medical Students: A Cross-Sectional Study

**DOI:** 10.7759/cureus.117939

**Published:** 2026-10-14

**Authors:** Patricio A Jiménez, Luz M Dressendörfer

**Affiliations:** 1 Department of Dermatology, Pontifical Catholic University of Ecuador, Quito, ECU

**Keywords:** acne vulgaris, epidemiology, facial cosmetic, instagram reels, lifestyle, logistic regression analysis, tiktok skincare trends, unsupervised drug use

## Abstract

Background and objectives: The self-directed use of acne treatment products is common among university students, particularly medical students. The factors associated with this practice, including exposure to acne-related content on social media, remain insufficiently understood. This study aimed to estimate the prevalence of self-directed acne treatment product use among medical students and identify factors associated with this practice, focusing on social media exposure, acne severity, and other potential predictors, using multivariable logistic regression to adjust for potential confounders.

Materials and methods: A descriptive cross-sectional study was conducted at a medical school during the 2024-2025 academic period. Students from the first to the tenth semester with acne completed a structured questionnaire. Associations were evaluated using bivariate analysis and binary logistic regression (Enter method). Odds ratios (ORs) and 95% confidence intervals (CIs) were estimated.

Results: Of 400 students approached, 360 met the eligibility criteria. After excluding four students who declined to participate and 46 who submitted incomplete questionnaires, 310 were included in the final analysis. The prevalence of self-directed use of acne treatment products was 38.4% (95% CI, 33.0-43.8). Viewing acne-related content on TikTok or Instagram Reels was significantly associated with this practice (OR = 2.52; 95% CI: 1.47-4.30; p = 0.001). Considering self-treatment as a self-care practice was also associated (OR = 2.26; 95% CI: 1.33-3.83; p = 0.002), as was greater acne severity (OR = 4.07; 95% CI: 1.55-10.65; p = 0.004).

Conclusions: Unsupervised use of acne treatment products was frequent among medical students. Exposure to acne-related content on TikTok and Instagram Reels, perceiving self-treatment as a self-care practice, and greater acne severity were independently associated with this behavior. Given the cross-sectional design and the limited assessment of social media exposure, these findings should not be interpreted as evidence of causal relationships.

## Introduction

Acne vulgaris is a chronic inflammatory disease of the pilosebaceous unit, which comprises the hair follicle, hair shaft, and sebaceous gland, and should not be regarded merely as a normal stage of the life cycle, as it is often colloquially perceived [1]. According to the Global Burden of Disease Study 2021, the global age-standardized prevalence rate of acne vulgaris was 3142.5 per 100000 population (95% uncertainty interval (UI): 2867.0-3500.6), while the age-standardized incidence rate was 1645.2 per 100000 population (95% UI: 1459.7-1878.2) [2,3]. Clinically, acne may present with comedones, papules, pustules, nodules, scars, and post-inflammatory hyperpigmentation [4]. Treatment is based on topical and systemic therapies, including antibiotics, retinoids, and hormonal agents.

Unsupervised use of acne treatment products is defined as the use of medications and/or dermocosmetic products without guidance from a healthcare professional for the purpose of acne treatment or management [5]. This phenomenon has increased in the context of the widespread availability and easy access to over-the-counter products. Several studies have reported a high prevalence of this practice among university students, particularly among medical students, who exhibit higher rates compared with students from other academic disciplines [6].

Multiple factors potentially associated with this practice have been described in the literature; however, few studies have performed analytical statistical approaches to evaluate these associations. Univariate analyses have suggested that medical students in more advanced academic semesters are more likely to engage in self-directed use of acne treatment products [7], indicating that dermatology training status may influence this behavior.

Likewise, more than half of individuals affected by acne believe that the condition can be improved through frequent facial cleansing [8], suggesting that acne may also be perceived as a condition whose pharmacological and non-pharmacological management is relatively simple. Similarly, more than half of medical students who self-prescribe acne treatment products consider this practice to be part of self-care [7], whereas others report engaging in this behavior to save time [9-11].

Moreover, the expansion of social media platforms such as Instagram Reels and TikTok, which disseminate short-form skincare and acne-related content, has transformed the way young individuals access and adopt therapeutic recommendations. Currently, there is a growing tendency for the public to seek medical information and advice through social media platforms [12]. Therefore, it is relevant to identify which factors are most strongly associated with unsupervised use of acne treatment products among medical students.

## Materials and methods

Study design and sampling

A descriptive cross-sectional study was conducted among 1315 medical students enrolled from the first to the tenth semester at the College of Medicine of Pontifical Catholic University of Ecuador, a large private university in Quito, Ecuador, during the 2024-2025 academic period. As the number of students with acne was unknown before recruitment, the sample size was calculated using the total enrolled student population as the finite population. Assuming an estimated prevalence of unsupervised use of acne treatment products of 52%, a 95% confidence level, and a 5-percentage-point margin of error, the minimum required sample size was 297 participants. Eligibility was subsequently assessed according to the predefined inclusion criteria. A non-probability quota sampling method was employed. Participants were recruited in classroom settings across academic semesters, with quotas established proportionally to the number of students enrolled in each semester. Figure 1 summarizes the participant selection process, including eligibility assessment, exclusions, and the final analytical sample. The study protocol was approved by the research ethics committee of the Pontifical Catholic University of Ecuador (protocol no. EO-047-2024).

**Figure 1:**
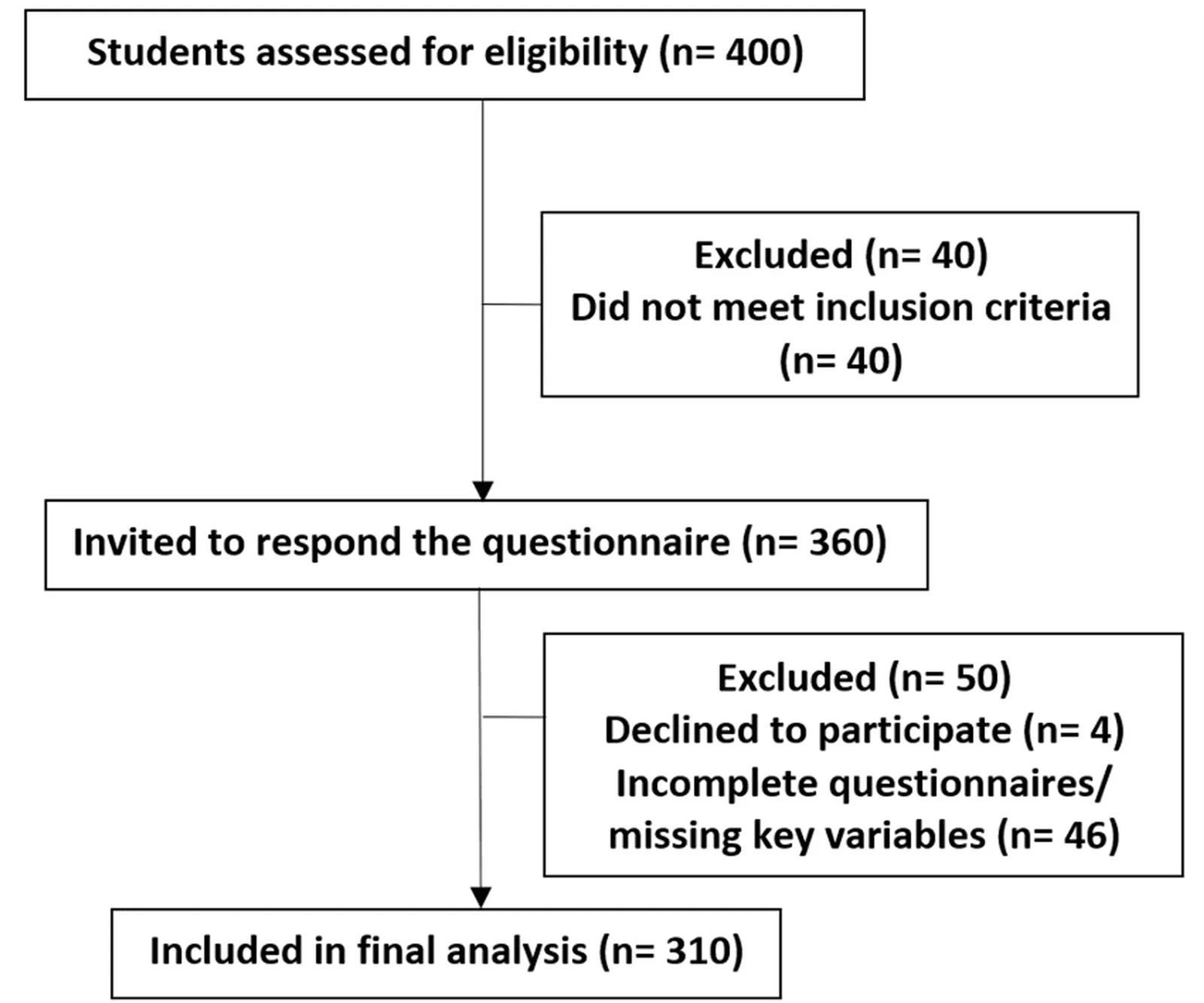
Flowchart of participant selection

Participants

Medical students aged 18-25 years with acne were included. Acne severity ranged from almost no lesions to very severe disease (Grade 1 to Grade 5) and was assessed independently by the two authors using the validated Global Acne Severity Scale (GEA Scale) [13]. Prior to data collection, evaluators underwent standardized training. Inter-rater reliability was assessed in a subset of 50 participants using Cohen’s kappa (κ) coefficient, demonstrating substantial agreement (κ= 0.78). Students beyond the tenth semester were excluded because survey completion could not be adequately supervised in that group.

Variables

We evaluated nine variables previously described as potentially associated with the unsupervised use of acne treatment products. The dependent variable was unsupervised use of acne treatment products, while the independent variables included sex, age, dermatology training status, acne severity grade, perceived treatment complexity, perception of self-treatment as a self-care practice, financial and time availability to access specialized care, ease of purchasing acne treatment products (in pharmacies and retail stores), and viewing acne-related content on TikTok/Instagram Reels. Further details regarding variable operationalization are provided in Appendix A.

Data sources

An author-designed online questionnaire (Appendix B) was developed and administered in person under supervision. The questionnaire included an informed consent section at the beginning, which participants were required to accept prior to participation. The recall period for the questionnaire items was the preceding three months.

Statistical methods

Binomial logistic regression analysis was performed to identify variables independently associated with the unsupervised use of acne treatment products and to control for potential confounding factors. The dependent variable was unsupervised use of acne treatment products, coded as a binary outcome (1= no, 2= yes). All independent variables were categorical and numerically coded prior to analysis. For regression modeling, categorical variables were entered as dummy variables, using the lowest coded category as the reference group.

Binary variables were coded as 1= no and 2= yes, including financial and time availability to access specialized care, considering self-treatment as a self-care practice, ease of purchasing acne treatment products, and viewing acne-related content on TikTok/Instagram Reels. Sex was coded as 1= female and 2= male. Dermatology training status was categorized as pre-dermatology training (first-fifth semesters) and post-dermatology training (sixth-tenth semesters), according to exposure to acne-related academic content.

Age was analyzed as a categorical variable (18-20 years, 21-23 years, and 24-25 years). Perceived treatment complexity was categorized as complicated, neutral, or easy. Due to the low frequency of severe acne cases, acne severity was regrouped into three categories (Grade 1, Grade 2, and Grade ≥3) to facilitate statistical analysis and interpretation of results.

Odds ratios (ORs) were interpreted relative to their respective reference categories. A total of 46 participants were excluded from the analysis due to missing data; therefore, only participants with complete data for the variables included in the analysis were retained. Variables were simultaneously entered into the multivariable binary logistic regression model using the Enter method, based on clinical relevance, epidemiological plausibility, and findings from the bivariate analysis. Model goodness of fit was assessed using the Hosmer-Lemeshow test and Nagelkerke’s R². Data were entered and analyzed using IBM SPSS Statistics software, version 25.0 (IBM Corp., Armonk, NY, USA). Statistical significance was defined as a two-tailed p-value < 0.05.

## Results

The minimum required sample size was 297 participants. Of the 400 students approached, 360 met the eligibility criteria. After exclusion of four students who declined to participate and 46 who submitted incomplete questionnaires, 310 participants were included in the final analysis (86.1% completion rate). Of these, 205 (66.1%) were female and 105 (33.9%) were male.

The prevalence of unsupervised use of acne treatment products was 110 (38.4%; 95% confidence interval (CI): 33.0-43.8), with topical administration being the most frequently reported route of use, 108 (90.8%). Dermocosmetic products were the most used category, 59 (49.5%), followed by medications, 48 (40.3%), and combined use of both product types, 12 (10.0%). Among dermocosmetic products, chemical exfoliants (salicylic acid, glycolic acid, and lactic acid) were the most frequently self-administered, 45 (69.2%), followed by sebum-regulating vitamins (niacinamide), 11 (16.9%), retinol-containing products, six (9.2%), physical exfoliants (sponges and micro-particle scrubs), two (3.0%), and other products, one (1.5%). Among medications, the most frequently used were retinoids (tretinoin, adapalene, isotretinoin), 35 (64.8%), followed by antibiotics (clindamycin, minocycline, doxycycline, azithromycin), 12 (22.2%), other antimicrobial agents (benzoyl peroxide, sulfur), four (7.4%), hormonal therapies (spironolactone, contraceptives), two (3.7%), and other medications, one (1.8%). Of the respondents, 174 (56.1%; 95% CI: 50.6-61.6) reported viewing acne-related content on TikTok/Instagram Reels.

A multivariable binomial logistic regression analysis using the Enter method was performed. Prior to model construction, multicollinearity among independent variables was assessed using the Variance Inflation Factor (VIF). All variables showed VIF values < 2 and tolerance values > 0.5, indicating no evidence of significant multicollinearity (Table 1).

**Table 1:** Multicollinearity tests (tolerance and variance inflation factor (VIF)). ^a^Dependent variable: unsupervised use of acne treatment products in the last three months. Multicollinearity was assessed using tolerance and the VIF. A VIF <5 and tolerance >0.20 were considered indicative of no substantial multicollinearity.

Coefficients^a^			
Model	Sig.	Collinearity Statistics	
		Tolerance	VIF
(Constant)	0.019	-	-
Sex	0.197	0.89	1.123
Age	0.675	0.563	1.777
Dermatology training status	0.582	0.551	1.816
Acne severity	0.002	0.94	1.064
Personal perception of self-treatment as a self-care practice	0.002	0.948	1.055
Perceived treatment complexity	0.681	0.942	1.062
Financial and time availability to access specialized medical care for acne	0.693	0.924	1.082
Ease of purchasing products (in pharmacies and stores) to treat acne	0.557	0.936	1.069
Viewing acne-related content on TikTok/Instagram Reels.	0.001	0.886	1.129

The descriptive characteristics of the study population, including absolute and relative frequencies according to unsupervised use of acne treatment products, are presented in Table 2. Viewing acne-related content on TikTok/Instagram Reels, considering self-treatment as a self-care practice, and greater acne severity were significantly associated with unsupervised use of acne treatment products. Crude and adjusted ORs with corresponding CIs and p-values are shown in Table 3.

**Table 2:** Characteristics of the study population according to unsupervised use of dermatologic products. df: degrees of freedom; p-values were calculated using Pearson’s chi-square test (χ²). p < 0.05 was considered statistically significant; * indicates statistically significant p-values.

Variable	Total (n=310), n (%)	No unsupervised use of products (n= 191), n (%)	Unsupervised use of products (n= 119), n (%)	Test statistic, χ² (df)	p-value
Sex				2.932 (1)	0.087
Female	206 (66.5%)	120 (62.8%)	86 (72.3%)	-	-
Male	104 (33.5%)	71 (37.2%)	33 (27.7%)	-	-
Age				0.891 (2)	0.641
18-20 years	141 (45.5%)	85 (44.5%)	56 (47.1%)	-	-
21-23 years	142 (45.8%)	91 (47.6%)	51 (42.9%)	-	-
24-25 years	27 (8.7%)	15 (7.9%)	12 (10.1%)	-	-
Dermatology training status				0.553 (1)	0.457
Pre-dermatology training (first – fifth semester)	193 (62.3%)	122 (63.9%)	71 (59.7%)	-	-
Post-dermatology training (sixth – tenth semester)	117 (37.7%)	69 (36.1%)	48 (40.3%)	-	-
Acne severity				12.774 (2)	0.002*
Grade 1	230 (74.2%)	153 (80.1%)	77 (64.7%)	-	-
Grade 2	55 (17.7%)	30 (15.7%)	25 (21.0%)	-	-
Grade 3 or higher	25 (8.1%)	8 (4.2%)	17 (14.3%)	-	-
Personal perception of self-treatment as a self-care practice				12.272 (1)	0.00046*
No	127 (41.0%)	93 (48.7%)	34 (28.6%)	-	-
Yes	183 (59.0%)	98 (51.3%)	85 (71.4%)	-	-
Perceived treatment complexity				0.292 (2)	0.864
Complicated	207 (66.8%)	128 (67.0%)	79 (66.4%)	-	-
Regular	85 (27.4%)	51 (26.7%)	34 (28.6%)	-	-
Easy	18 (5.8%)	12 (6.3%)	6 (5.0%)	-	-
Financial and time availability to access specialized medical care				0,0004 (1)	0.983
No	94 (30.3%)	58 (30.4%)	36 (30.3%)	-	-
Yes	216 (69.7%)	133 (69.6%)	83 (69.7%)	-	-
Ease of purchasing products (in pharmacies and stores) to treat acne				1.632 (1)	0.201
No	38 (12.3%)	27 (14.1%)	11 (9.2%)	-	-
Yes	272 (87.7%)	164 (85.9%)	108 (90.8%)	-	-
Viewing acne-related content on TikTok/Instagram Reels				16.398 (1)	0.000051*
No	136 (43.9%)	101 (52.9%)	35 (29.4%)	-	-
Yes	174 (56.1%)	90 (47.1%)	84 (70.6%)	-	-

**Table 3:** Binomial logistic regression analysis of the independent variables significantly associated with self-medication, with crude and adjusted estimates. ref: reference category; df: degrees of freedom. P-values were calculated using the Wald test; p < 0.05 was considered statistically significant; * indicates statistically significant p-values.

Variable	Category	Wald (df)	Crude OR (95% CI)	p-value	Wald (df)	Adjusted OR (95% CI)	p-value
Sex	Female	-	1 (ref)	-	-	-	-
	Male	2.914 (1)	0.65 (95% CI: 0.39–1.07)	0.088	1.049 (1)	0.74 (95% CI: 0.42-1.31)	0.306
Age	18-20 years	-	1 (ref)	-	-	-	-
	21-23 years	0.434 (1)	0.84 (95% CI: 0.51-1.37)	0.51	1.295 (1)	0.65 (95% CI: 0.31-1.36)	0.255
	24-25 years	0.210 (1)	1.21 (95% CI: 0.52-2.78)	0.547	0.009 (1)	0.95 (95% CI: 0.33-2.72)	0.923
Dermatology training status	Pre- dermatology training (1st – 5th semester)	-	1 (ref)	-	-	-	-
	Post- dermatology training (6th – 10th semester)	0.553 (1)	1.19 (95% CI: 0.74-1.91)	0.457	0.705 (1)	1.38 (95% CI: 0.64-2.99)	0.401
Acne severity	Grade 1	-	1 (ref)	-		-	-
	Grade 2	2.739 (1)	1.66 (95% CI: 0.91–3.01)	0.098	1.940 (1)	1.58 (95% CI: 0.82-3.02)	0.164
	Grade 3 or higher	10.203 (1)	4.22 (95% CI 1.75–10.22)	0.001	8.208 (1)	4.07 (95% CI: 1.55-10.65)	0.004*
Personal perception of self-treatment as a self-care practice	No	-	1 (ref)	-	-	-	-
	Yes	12.012 (1)	2.37 (95% CI: 1.45-3.86)	0.001	9.242 (1)	2.26 (95% CI: 1.33-3.83)	0.002*
Perceived treatment complexity	Complicated	-	1 (ref)	-	-	-	-
	Regular	0.277 (1)	1.33 (95% CI: 0.45-3.89)	0.599	0.419 (1)	1.47 (95% CI: 0.45-4.81)	0.517
	Easy	0.164 (1)	1.23 (95% CI: 0.44-3.42)	0.686	0.095 (1)	1.19 (95% CI: 0.38-3.71)	0.758
Financial and time availability to access specialized medical care	No	-	1 (ref)	-	-	-	-
	Yes	0.0004 (1)	1.00 (95% CI: 0.61-1.65)	0.983	1.806 (1)	1.13 (95% CI: 0.64-1.98)	0.666
Ease of purchasing products (in pharmacies and stores) to treat acne	No	-	1 (ref)	-	-	-	-
	Yes	1.609 (1)	1.61 (95% CI: 0.77-3.39)	0.205	0.309 (1)	1.26 (95% CI: 0.54-2.93)	0.579
Viewing acne-related content on TikTok/ Instagram Reels.	No	-	1 (ref)	-	-	-	-
	Yes	15.965 (1)	2.69 (95% CI 1.65-4.37)	0.00065	11.402 (1)	2.52 (95% CI: 1.47-4.30)	0.001*

Model fit was assessed using the Hosmer-Lemeshow test (χ² = 8.931; p= 0.348) (Table 4), indicating no evidence of substantial disagreement between observed and predicted values. The model yielded a Nagelkerke R² of 0.177, indicating a moderate explanatory capacity for the outcome in the context of multifactorial behavioral phenomena.

**Table 4:** Nagelkerke's R-squared and Hosmer and Lemeshow goodness-of-fit test df: degrees of freedom

Model Summary			
Step	-2 Log Likelihood	Cox and Snell R-squared	Nagelkerke's R-squared
1	396.653	0.130	0.177
Hosmer and Lemeshow test			
Step	Chi-square	df	Significance
1	8.931	8	0.348

## Discussion

Despite the many factors that may contribute to the use of unsupervised acne treatment products among young medical students, the relative strength and significance of these associations have not been thoroughly investigated. Using a binomial logistic regression model, we evaluated variables previously described in the literature and identified those independently associated with this practice. Self-prescription for acne represents a complex behavioral phenomenon that, as demonstrated both in our study and in previous reports [7,10], is relatively common among medical students. Topical therapies containing active ingredients were the most frequently used products, likely due to their accessibility and ease of use, consistent with findings from previous studies [14,15].

Although dermocosmetic products are generally perceived as safer and more accessible than pharmacological treatments, their unsupervised use may still result in inappropriate product selection, excessive or incompatible combinations of active ingredients, skin irritation, and impairment of the skin barrier. The beneficial effects of products containing alpha hydroxy acids are well established, even at the low concentrations commonly found in over-the-counter formulations. However, appropriate indications and precautions for their use are often overlooked in the absence of adequate guidance. Many of these acids are not recommended for individuals with sensitive skin, and regular daily sun protection is recommended during their use [16]. Similar concerns apply to other commonly self-administered products, including retinol and niacinamide-containing formulations. Furthermore, self-directed use of these products may contribute to delays in seeking professional dermatological care, particularly among individuals who repeatedly attempt over-the-counter regimens before consulting a specialist.

Current evidence indicates that dermocosmetics may play an important role in acne management when appropriately selected and incorporated into individualized treatment plans. However, reviews and expert consensus statements emphasize that patient education and professional guidance remain essential to optimize tolerability, adherence, and therapeutic outcomes [17-19]. The use of social media, particularly TikTok and Instagram Reels, as a source of acne-related information was reported by more than half of the study participants, reflecting a broader trend in which approximately 83% of young individuals seek medical information online [20].

Our findings suggest that exposure to acne-related content on social media may be associated with a higher likelihood of self-directed use of acne treatment products, consistent with findings from recent small-sample studies [21]. This may represent a relevant concern, as much of the information disseminated on these platforms does not originate from healthcare professionals. For example, a study evaluating acne treatment content on YouTube found that 63 (65.6%) of the videos reflected the perspectives of patients or bloggers, whereas only 14 (4.6%) featured dermatologists [22]. Even when information is provided by specialists, potential risks should still be considered, as financial incentives from pharmaceutical companies to promote products on social media may introduce conflicts of interest, influence the doctor-patient relationship, and encourage the use of unprescribed products [23].

In addition, we found that considering self-treatment as a self-care practice was significantly associated with unsupervised product use. This association may be partially explained by the perception of increased autonomy and control that can develop during medical training, potentially leading students to justify unguided product use as a rational form of self-care [24]. Our findings also suggest that greater acne severity may contribute to self-directed product use, possibly reflecting the psychological burden, aesthetic distress, and urgency to improve the condition among individuals with acne [25].

However, several limitations should be considered when interpreting these findings. First, the study was conducted at a single private medical school, which may limit the generalizability of the results to public universities, nonmedical students, and other academic or geographic settings. The findings also may not fully reflect the experiences of students with limited access to social media or from different socioeconomic backgrounds. Multicenter studies involving public and private institutions, students from diverse academic disciplines, and populations with varying levels of digital access are warranted to assess the generalizability of these findings.

Second, unsupervised use of acne treatment products was assessed as a combined outcome without distinguishing between medications and dermocosmetic products. These categories differ in composition, regulatory status, and potential risks; therefore, their combined assessment may obscure clinically relevant differences in use patterns and associated consequences. Future studies should assess these categories separately to provide a more detailed characterization of unsupervised treatment practices. Furthermore, social media exposure was assessed using a single dichotomous (yes/no) item, which did not capture the frequency or intensity of exposure or the specific types of acne-related content encountered. Consequently, the study could not characterize social media exposure in greater detail or determine which types of content may be associated with decisions regarding acne treatment product use.

Third, the questionnaire was not formally validated, which may have affected the accuracy of the measurements. In particular, the perception of self-treatment as a self-care practice was assessed using a single item that may not fully capture the complexity of this concept. Although this item assessed participants’ beliefs rather than their actual treatment practices, conceptual overlap with the study outcome cannot be excluded. In addition, reliance on self-reported data may have introduced recall or social desirability bias.

Finally, although the variables included in the analysis were selected based on their relevance to the research question and the information collected, unmeasured factors may have influenced the observed associations. Therefore, residual confounding cannot be excluded. Moreover, the cross-sectional design precludes establishing temporal relationships between the exposures and the outcome; thus, the observed associations should not be interpreted as causal, and reverse associations cannot be ruled out.

## Conclusions

Unsupervised use of acne treatment products was frequent among medical students. Exposure to acne-related content on TikTok and Instagram Reels, perceiving self-treatment as a self-care practice, and greater acne severity were independently associated with this behavior. Given the cross-sectional design and the limited assessment of social media exposure, these findings should not be interpreted as evidence of causal relationships.

## Appendices

Appendix A 

**Table 5:** Operationalization of variables

Variable	Definition	Dimension	Indicator	Escale	Type
Age	Age in years based on date of birth	-	Relative frequencies	18-20 21-23 24-25 Other age	Ordinal qualitative
Sex	Chromosomally determined biological condition	-	Relative frequencies	Male Female	Nominal qualitative
Acne severity	Clinical acne severity assessed by trained evaluators using the Global Acne Severity Scale (GEA Scale).	-	Relative frequencies	Clear. No lesions: Residual pigmentation and erythema may be seen Grade 1/ Almost clear. Almost no lesions: A few scattered open or closed comedones and very few papules. Grade 2/ Mild: Easily recognizable: less than half of the face is involved. A few open or closed comedones and a few papules and pustules. Grade 3/ Moderate: More than half of the face is involved. Many papules and pustules, many open or closed comedones. One nodule may be present. Grade 4/ Severe: Entire face is involved, covered with many papules and pustules, open or closed comedones and rare nodules Grade 5/ Very severe: Highly inflammatory acne covering the face with presence of nodules.	Ordinal qualitative
Unsupervised use of acne treatment products	Use of medications and/or dermocosmetic products without guidance from a healthcare professional for the purpose of acne treatment or management.	-	Relative frequencies	Yes No	Dichotomous qualitative
Route of administration for self-prescribed products	Way drugs used to treat acne reach the body.	-	Relative frequencies	Topical (creams, ointments, serums, lotions) Oral (capsules, tablets, pills) Other: Parenteral, inhaled, etc.	Nominal qualitative
Product used for acne treatment	Category to which the product used for acne treatment belongs. Medications were defined as active ingredients with an explicit therapeutic indication for acne treatment. Dermocosmetic products were defined as active ingredients intended exclusively for skin care and not requiring prescription-based dispensing.	-	Relative frequencies	•Medications (antibiotics, retinoids, hormonal therapies) •Dermocosmetic products (chemical and physical exfoliants, sebum-regulating agents, retinol-containing products) •Both (medications and dermocosmetic products)	Nominal qualitative
Medication used for acne treatment	Pharmacological group to which the self-prescribed medication belongs.		Relative frequencies	Topical/oral retinoids (tretinoin, adapalene, isotretinoin) Topical/oral antibiotics (clindamycin, minocycline, doxycycline, azithromycin) Hormonal therapies (spironolactone, contraceptives) Other topical/oral antimicrobials (benzoyl peroxide, sulfur) Other medications	Nominal qualitative
Dermocosmetic used for acne treatment:	Group to which the self-prescribed dermocosmetic belongs.		Relative frequencies	Chemical exfoliants (salicylic acid, glycolic acid, lactic acid) Physical exfoliants (microbead scrubs, sponges) Sebum-regulating agents (niacinamide) Retinol-containing products Other dermocosmetics	Nominal qualitative
Ease of purchasing products to treat acne	Capacity to physically access (in pharmacies and stores) products available for sale to treat acne.	-	Relative frequencies	Yes No	Dichotomous qualitative
Perceived treatment complexity	Perception of the difficulty of treating acne on one's own.	-	Relative frequencies	Complicated Regular Easy	Ordinal qualitative
Personal perception of self-treatment as a self-care practice	Participant’s perception of whether treating acne independently constitutes a form of self-care practice.	-	Relative frequencies	Yes No	Dichotomous qualitative
Viewing acne-related content on TikTok/Instagram Reels.	Viewing content or short videos that include information about acne on these platforms within the last 3 months.	-	Relative frequencies	Yes No	Dichotomous qualitative
Specialist accessibility	Students’ financial and time availability to access specialized medical care for acne.	-	Relative frequencies	Yes No	Dichotomous qualitative
Dermatology training status	Dermatology training status according to the medical curriculum and exposure to acne-related academic content.	-	Relative frequencies	Pre-dermatology training (1st – 5th semester) Post-dermatology training (6th – 10th semester)	Ordinal qualitative

Appendix B

Questionnaire

1.      Age in completed years

a.      18- 20

b.      21-23

c.       24-25 

d.      Other age

2.      Sex

a.      Male

b.      Female

3.      Current semester of study

a.      First-fifth semester

b.      Sixth-tenth semester

4.      What degree of acne do you currently have, according to the evaluator’s assessment?

a.      Clear. No lesions: Residual pigmentation and erythema may be seen

b.      Grade 1/ Almost clear. Almost no lesions: A few scattered open or closed comedones and very few papules.

c.       Grade 2/ Mild: Easily recognizable: less than half of the face is involved. A few open or closed comedones and a few papules and pustules.

d.      Grade 3/ Moderate: More than half of the face is involved. Many papules and pustules, many open or closed comedones. One nodule may be present.

e.      Grade 4/ Severe: Entire face is involved, covered with many papules and pustules, open or closed comedones, and rare nodules

f.        Grade 5/ Very severe: Highly inflammatory acne covering the face with presence of nodules.

5.      In the last three months, have you used medications and/or dermocosmetics without the guidance of a healthcare professional for the purpose of treating acne?

a.      Yes

b.      No

6.      What is the route of administration for the medications you use to treat your acne?

a.      Topical (creams, ointments, serums, lotions)

b.      Oral (capsules, tablets, pills)

c.       Other: Parenteral, inhaled, etc.

7.      Which group of products do you use most frequently, without professional supervision, for the treatment of your acne?

a.      Medications (antibiotics, retinoids, hormonal therapies)

b.      Dermocosmetic products (chemical and physical exfoliants, sebum-regulating agents, retinol-containing products)

c.       Both (medications and dermocosmetic products)

8.      If the answer to Question 7 was medications: Which group of medications was most frequently used, without the guidance of a healthcare professional, to treat your acne?

a.       Topical/oral retinoids (tretinoin, adapalene, isotretinoin)

b.       Topical/oral antibiotics (clindamycin, minocycline, doxycycline, azithromycin)

c.       Hormonal therapies (spironolactone, contraceptives)

d.       Other topical/oral antimicrobials (benzoyl peroxide, sulfur)

e.       Other medications

9.      If the answer to Question 7 was dermocosmetics: Which group of dermocosmetics was most frequently used, without the guidance of a healthcare professional, to treat your acne?

a.      Chemical exfoliants (salicylic acid, glycolic acid, lactic acid)

b.      Physical exfoliants (microbead scrubs, sponges)

c.       Sebum-regulating agents (niacinamide)

d.      Retinol-containing products

e.      Other dermocosmetics

10.  Do you have easy access (in pharmacies and stores) to products available for sale to treat acne?

a.      Yes

b.      No

11.  How complicated do you consider it to be to treat acne on your own?

a.      Complicated

b.      Regular

c.       Easy

12.  Do you consider treating acne on your own to be part of self-care?

a.      Yes

b.      No

13.  Do you watch TikTok or Instagram Reels videos about acne?

a.      Yes

b.      No

14.  Do you have the financial capacity and time to access specialized medical care for acne?

a.      Yes

b.      No
